# Supplementary material for: The Characteristics of Badminton-Related Pain in Pre-Adolescent and Adolescent Badminton Players
Source: Children (Basel). 2023 Sep 2;10(9):1501. doi: 10.3390/children10091501 (PMC10530166; doi:10.3390/children10091501)
Supplement: Supplementary file 1 [file children-10-01501-s001.zip › children-2522503-Supplementary Materials.pdf]

Translation: Injury and Pain Questionnaire

Survey on Sports Medical Science Support for Junior Athlete

1.Date:______________2.City___________3.Age:__________4.Gender (Male・Female) 5.Grade_____6 the age to start competitive_____7.Dominant side (Right・Left) 8. Training time (last year) (about hours/day, about days/week, about weeks/year)

Please select the number of the place where you have had pain or injury that affects your daily life or badminton. Write it from 1-25 in the figure below (It doesn't matter how many you write (left or right)).

( )

In the places listed above, write the number of the part where you consulted with your parents or coach.

( )

In the places listed above, please write the number of the part where you actually required medical care such as a hospital.

( )

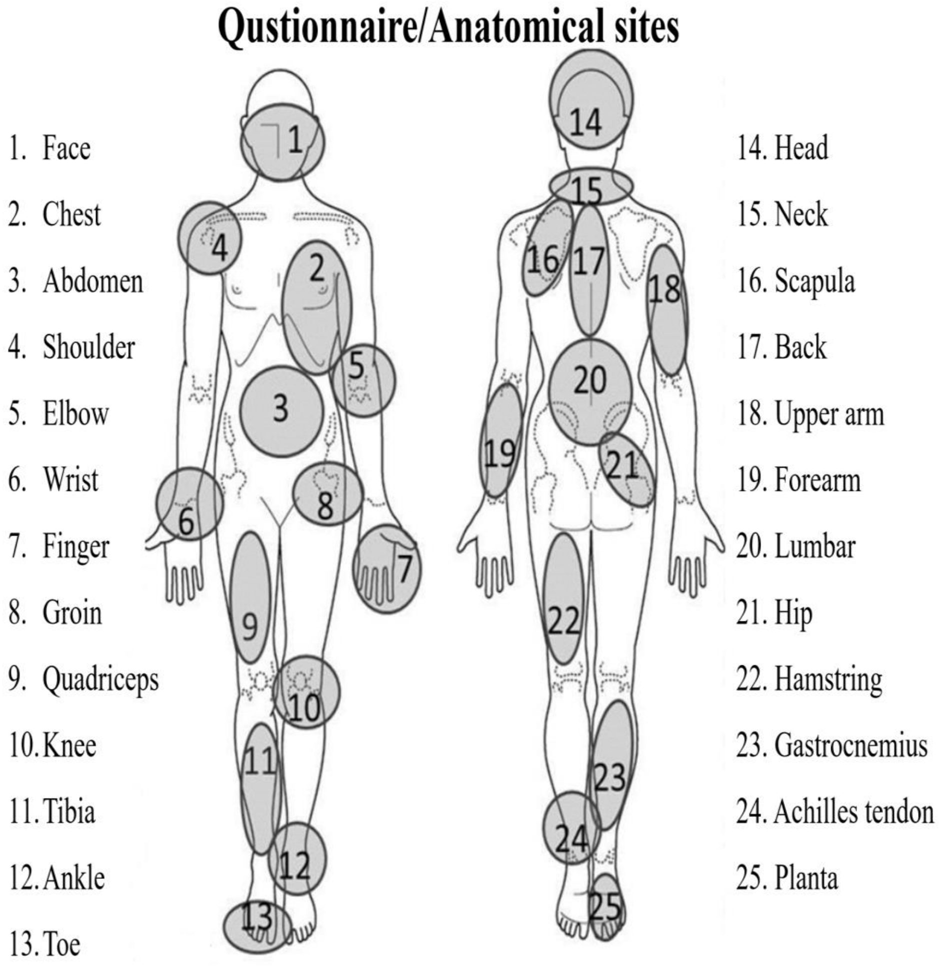

Figure S1. Anatomical regions image

Translation: Injury and Pain Questionnaire

1.Upper body (Figures: 1-7 front, 14-20 back). Is there a place where there was an injury or pain? If there is, please fill in the form below.

Items (Right・left, anatomical site, injury nature or pain, age, the status that happened, current status)

Example (Right・left, shoulder, pain, 12 years and 2 months old, during the training, the pain eliminated in about a month); (Right・left, lower back, lumbar spondylosis, 11 years and 8 months old, during the training, current pain)

A (   ), B (   ), C (   ), D (   ), E (   ).

2.Lower body (Figures: 8-13 front, 21-25 back). Is there a place where there was an injury or pain? If there is, please fill in the form below.

Items (Right・left, anatomical site, injury nature or pain, age, the status that happened, current status)

Example (Right・left, shoulder, pain, 12 years and 2 months old, during the training, the pain eliminated in about a month); (Right・left, lower back, lumbar spondylosis, 11 years and 8 months old, during the training, current pain)

A (   ), B (   ), C (   ), D (   ), E (   ).

3. Do you warm up at the beginning of practice? (Yes   No).

If yes, please write the time of warm up (   ) minutes.

4 Do you cool down at the beginning of practice? (Yes   No).

If yes, please write the time of warm up (   ) minutes.

Thank you very much.
